# Molecular cytogenetic identification of new wheat-rye 6R, 6RS, and 6RL addition lines with resistance to stripe rust and powdery mildew

**DOI:** 10.3389/fpls.2022.992016

**Published:** 2022-08-19

**Authors:** Tianheng Ren, Zixin Sun, Yuling Hu, Zhenglong Ren, Feiquan Tan, Peigao Luo, Zhi Li

**Affiliations:** ^1^State key Laboratory of Crop Gene Exploration and Utilization in Southwest China, Sichuan Agricultural University, Chengdu, China; ^2^College of Agronomy, Sichuan Agricultural University, Chengdu, China

**Keywords:** *Triticum aestivum*, *Secale cereale*, chromosome addition, resistance, genetic resource

## Abstract

Stripe rust and powdery mildew are devastating diseases that have severe effects on wheat production. Introducing resistant genes/loci from wheat-related species into the wheat genome is an important method to improve wheat resistance. Rye (*Secale cereale* L.) is a cross-pollinating plant and is the most important related species for wheat genetic improvement. In this study, we developed three 6RS ditelosomic addition lines, three 6RL ditelosomic addition lines, and two 6R disomic addition lines by crossing common wheat cultivar Chuannong 25 and rye inbred line QL2. The chromosome composition of all new lines was confirmed by non-denaturing fluorescence *in situ* hybridization (ND-FISH) and molecular marker analyses. Disease responses to different *Puccinia striiformis* f. sp. *tritici* (*Pst*) races and *Blumeria graminis* f. sp. *tritici* (*Bgt*) isolates and cytogenetic analysis showed that the resistance of the new lines was derived from the rye chromosome 6R of QL2, and both arms (6RS and 6RL) may harbor resistance genes against *Pst* and *Bgt*. These new lines could be used as a promising bridging parent and valuable genetic resource for wheat disease resistance improvement.

## Introduction

Wheat (*Triticum aestivum* L.) is one of the most important food crops in the world and provides ~20% of the protein and calories for human beings (Chaves et al., 2013). However, stripe rust (caused by *Puccinia striiformis* f. sp. *tritici*, *Pst*) and powdery mildew (*Blumeria graminis* f. sp. *tritici*, *Bgt*) are typically devastating diseases and are very detrimental to wheat production in subtropical agricultural zones (Duveiller et al., 2007). Stripe rust and powdery mildew result in the loss of ~5% of production. However, in some of the most prevalent areas, stripe rust and powdery mildew can increase the loss to 20%–30% of production (Ren et al., 2009; Chen et al., 2014). For example, serious outbreaks of stripe rust resulted in a 25% yield loss in the United States from 1958 to 2014 (Chen, 2005, 2014). In China, stripe rust is epidemic almost every year and causes significant yield losses (Wan et al., 2007; Ren et al., 2022). In recent years, with the change in global climate, powdery mildew has affected wheat production more (Huo et al., 2002). For example, in 1990 and 1991, more than 12 million hectares of wheat were attacked by *Bgt* in China, causing 1.4 million and 0.7 million tons of yield losses, respectively (Hou et al., 2000).

Breeding disease-resistant wheat cultivars represent the most economical, environmentally friendly, and effective control measures at present. Related species of wheat, such as rye (*Secale cereale*; Schlegel and Korzun, 1997), *Haynaldia villosa* (Chen et al., 1995), *Elytrigia intermedium* (Luo et al., 2009), and *Agropyron cristatum* (Wang et al., 2022), contain a large number of resistance genes and have been used in wheat disease resistance breeding for a long time. Among them, the most valuable related species is rye (Schlegel and Korzun, 1997; Ren et al., 2022). Disease-resistance genes for *Pst* and *Bgt,* such as *Yr9*, *Pm8*, and *Pm17,* were introduced into the wheat genome *via* T1RS·1BL or T1RS·1AL translocation (Mater et al., 2004; Mago et al., 2005). However, due to the changes in the prevalent pathogens, these resistance genes were overcome by new pathogens at the end of the last century (Ren et al., 2009). In the past 10 years, several new 1RS chromosomes have been introduced into the wheat genome and have exhibited resistance to the new *Pst* and *Bgt* pathogens (Ren et al., 2017a, 2018, 2022; Han et al., 2020). Additionally, 2R, 4R, 5R, 6R, and 7R chromosomes are also introduced into the wheat genome in the form of chromosome translocation, addition, and substitution, and several of these newly developed lines exhibit resistance to stripe rust or powdery mildew (An et al., 2015, 2019; Li et al., 2016; Schneider et al., 2016; Ren et al., 2017b, 2020; Johansson et al., 2020; Han et al., 2022).

In this study, eight new addition lines, including three 6RS ditelosomic addition lines, three 6RL ditelosomic addition lines, and two 6R disomic addition lines, were developed and selected from a cross between the rye inbred line QL2 and wheat cultivar CN25. All eight addition lines showed high resistance to both *Pst* and *Bgt*. The results indicated that both the long arm and short arm of QL2 may contain resistance genes for *Pst* and *Bgt*. These eight new lines could represent valuable genetic resources for wheat disease resistance breeding programs in the future.

## Materials and methods

### Plant materials

Chuannong 25 (CN25) is a high-yield wheat cultivar that was approved by the Sichuan Provincial Variety Examination and Committee in 2007 and has been widely planted in southwestern China. The pure genetic stocks of CN25 used in this study were bred by single-spike descent over several generations. Rye Qinling was collected by our lab in the 1990s. An inbred line of Qinling rye, QL2, was used in this study. CN25 was crossed directly with QL2 to produce hybrid F_1_ seeds. Then, the F_1_ seedlings were treated with 0.05% colchicine plus 3% dimethyl sulfoxide for 8 h to produce the amphidiploid (C_1_). The C_1_ plants were backcrossed with wheat parent CN25 twice to produce the BC_2_F_1_ plants. From now on, only the plants that showed resistance to diseases in the field were harvested and reproduced in the generations BC_2_F_1_ to BC_2_F_4_. In the generation BC_2_F_5_, the chromosome composition of the plants was examined by non-denaturing fluorescence *in situ* hybridization (ND-FISH), and then, the plants were transplanted into the field. In this generation, several plants with rye 6R chromosomes were identified. The seeds of these plants were harvested and reproduced by selfing. In BC_2_F_6_, 50 seeds were randomly selected from each line and cultured in the lab first, and the chromosome composition of the plants was determined by ND-FISH. Then, all surviving plants were transplanted into the field. From BC_2_F_6_ to BC_2_F_8_, 6R disomic addition plants, 6RS ditelosomic addition plants, and 6RL ditelosomic addition plants were identified.

### Cytogenetic and molecular analyses

ND-FISH was used to identify the chromosome composition of the plant materials. Five oligonucleotide probes, Oligo-pSc119.2-1, Oligo-pTa535-1, Oligo-Ku, Oligo-pSc200, and Oligo-pSc250, were used in this study (Tang et al., 2014; Ren et al., 2019). The sequences and chromosomes that could be identified by these five probes are listed in Table 1. The wheat and rye chromosomes could be accurately distinguished by the combination of these five probes in one cell. Moreover, the centromeric-specific probe Oligo-CCS1, rye centromeric-specific probe Oligo-PAWRC.1, and telomere-specific probe Oligo-Telo were mixed and used in another ND-FISH experiment (Cuadrado and Jouve, 2010; Tang et al., 2014). Cell images were captured using an epifluorescence microscope (model BX51, Olympus, Center Valley, PA, United States) equipped with a cooled charge-coupled device camera and operated with the software program HCIMAGE Live (version 2.0.1.5, Hamamatsu Corp., Sewickley, PA, United States). The processes of sample preparation (root tips), probe labeling, and ND-FISH were performed according to Tang et al. (2014) and Ren et al. (2019). Molecular markers specific to 6RL and 6RS were also used to confirm the chromosome. The genomic DNA of the plant materials was isolated from young leaves using the surfactant cetyltrimethylammonium bromide (CTAB; Doyle and Doyle, 1987). Two primer pairs, KU88 and KU291, which were specific for the rye 6RS chromosome arm, and two primer pairs, KU86 and KU153, which were specific for the rye 6RL chromosome arm, were used (Qiu et al., 2016; Table 2). These primers can detect rye 6RS or 6RL chromosome arms in the wheat genetic background and can amplify specific ~400-bp fragment bands (Qiu et al., 2016). PCR and electrophoresis were performed according to Qiu et al. (2016).

**Table 1:** The sequences of the oligonucleotide probes for ND-FISH used in this study.

Probes	Chromosome or location	Sequences
Oligo-pSc119.2-1	4A, 5A, 1B-7B, 2D, 3D, 4D	6-FAM-5′-ccgttttgtggactattactcaccgctttggggtcccatagctat-3′
Oligo-pTa535-1	1A, 2A, 3A, 4A, 6A, 7A, 3B, 6B, 7B, 1D-7D	Tamra-5′-aaaaacttgacgcacgtcacgtacaaattggacaaactctttcggagtatcagggtttc-3′
Oligo-Ku	1R-7R	Tamra-5′-gatcgagacttctagcaataggcaaaaatagtaatggtatccgggttcg-3′
Oligo-pSc200	1R-7R	Tamra-5′-ctcacttgctttgagagtctcgatcaattcggactctaggttgatttttgtattttct-3′
Oligo-pSc250	1R-7R	Tamra-5′-tgtgttgttcttggacaaaacaatgcataccatctcttctac-3′
Oligo-CCS1	Wheat and rye centromere	6-FAM-5′-ccgtttgatagaggcaaaggtgtcccgtcttttgatgaga-3′
Oligo-PAWRC.1	Rye centromere	6-FAM-5′-ccgtttgatagaggcaaaggtgtcccgtcttttgatgaga-3′
Oligo-Telo	Telomere	Tamra-5′-tttagggtttagggtttaggg-3′

Cuadrado and Jouve (2010), Tang et al. (2014), Ren et al. (2019).

**Table 2:** The sequences of the specific molecular markers for PCR used in this study.

Markers	Forward (5′–3′)	Reverse (5′–3′)	Chromosome
KU88	caggatatcccacaacacaaga	atgggttgtatttgccgaaa	6RS
KU291	gagactacccgtcgaaggac	ggggcttcatcgacaatcta	6RS
KU86	acagccaagctcaaagtggt	tcagtgcagacggtgatagc	6RL
KU153	tggaacttccctttgaatgc	tggaagaaatgtgcagataaaca	6RL

Qiu et al. (2016).

### Stripe rust and powdery mildew tests

The *Pst* races CYR32 (virulent to *Yr1, 2, 3, 4, 6, 7, 8, 9, 17, 25, 27, 28, 31, 32, 43, 44, A, Alba, Cle, Gaby, Res, SD, SO, Exp2, SK*, and *SP*), CYR33 (virulent to *Yr1, 2, 3, 4, 6, 7, 8, 9, 17, 25, 28, 31, 32, A*, and *Su*), and CYR34 [virulent to *Yr1, 2, 3, 4, 6, 7, 8, 9, 10, 17, 19, 24(=26), 25, 27, 28, 31, 32, 43, 44, Exp2, SP, A*, and *Sk*] were used in this experiment (Ren et al., 2022). These three *Pst* races were considered the most virulent and frequent *Pst* races occurring in China and were used in stripe rust resistance tests at the seedling stage (Ren et al., 2022). The inoculation of *Pst* on wheat seedlings was performed according to Ren et al. (2020). Two *Bgt* isolates, E20 and No. 15, were used in the powdery mildew resistance tests at the seedling stage. Both *Bgt* isolates have been virulent to many newly released cultivars in China in recent years (Ren et al., 2017a; Yang et al., 2021). The inoculation of *Bgt* on wheat seedlings was performed according to Yang et al. (2021). The three *Pst* races were provided by the Plant Protection Institute, Gansu Academy of Agricultural Sciences, China. The *Bgt* isolate E20 was provided by the Department of Plant Protection, Sichuan Agricultural University. The *Bgt* isolate No. 15 was collected by our lab in Ya’an City, Sichuan, China (Ren et al., 2018). The inoculation of *Pst* and *Bg*t on wheat seedlings was performed with three replications in the greenhouse. In addition, the disease resistance of all materials identified in this study was tested in the field under severe natural *Pst* and *Bgt* infection at the Qionglai Research Station of Sichuan Agricultural University in Cheng Plain, Southwest China (30°25′N, 103°28′E) from 2020 to 2022. The field experiments were performed according to the methods described previously (Ren et al., 2020, 2022). The wheat parent CN25, rye parent QL2, wheat cultivar Mianyang 11, and RT1104-1 were served as controls. Stripe rust reactions were scored at the grain filling stage as infection types (ITs) based on a 0–9 scale as described by Wan et al. (2004) and Ren et al. (2022). Powdery mildew reactions were scored at the heading stage as ITs based on a 0–4 scale as described by Xie et al. (2004) and Ren et al. (2020).

## Results

### Development of disomic addition lines

**Figure 1:**
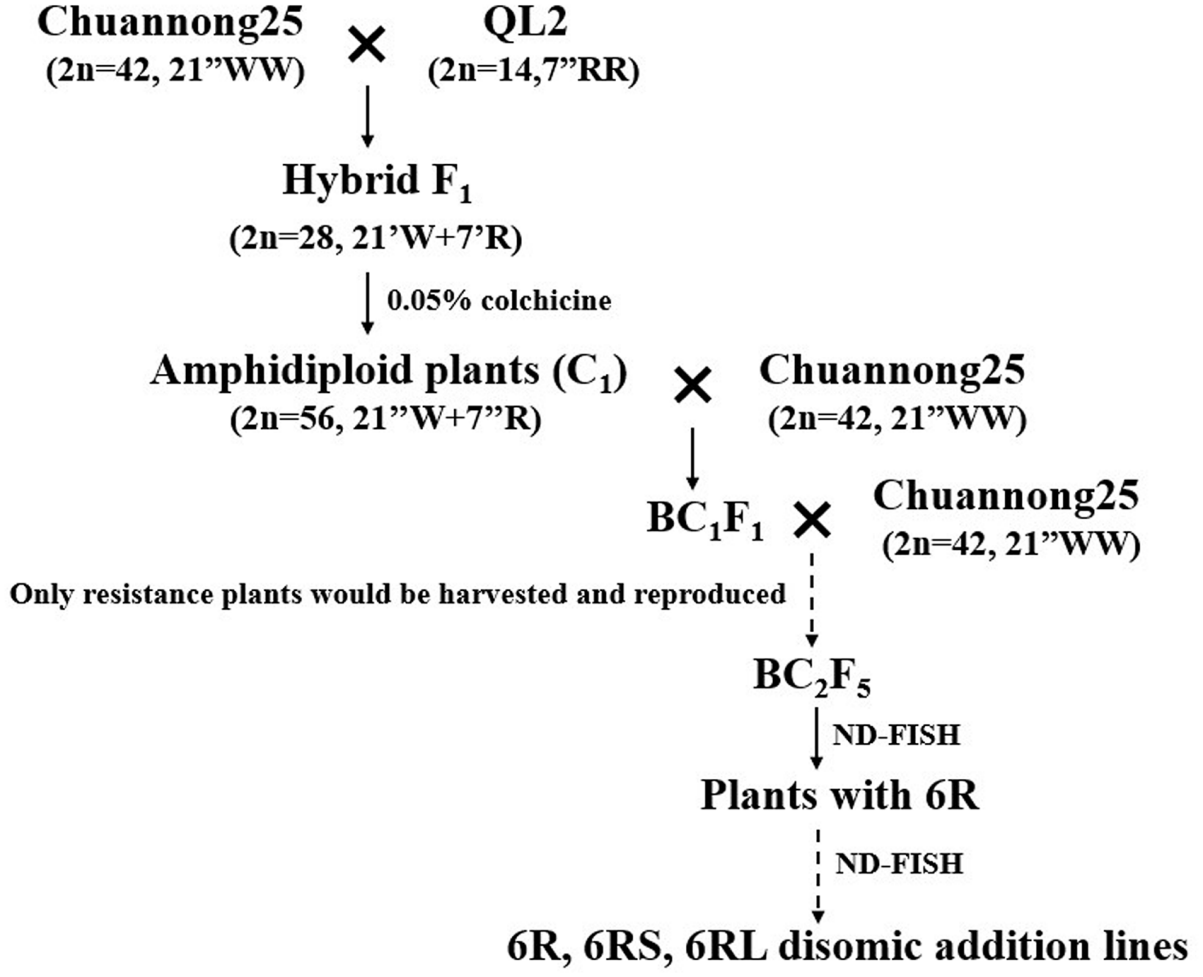
The pedigree of the new 6R, 6RS, and 6RL addition lines.

**Figure 2:**
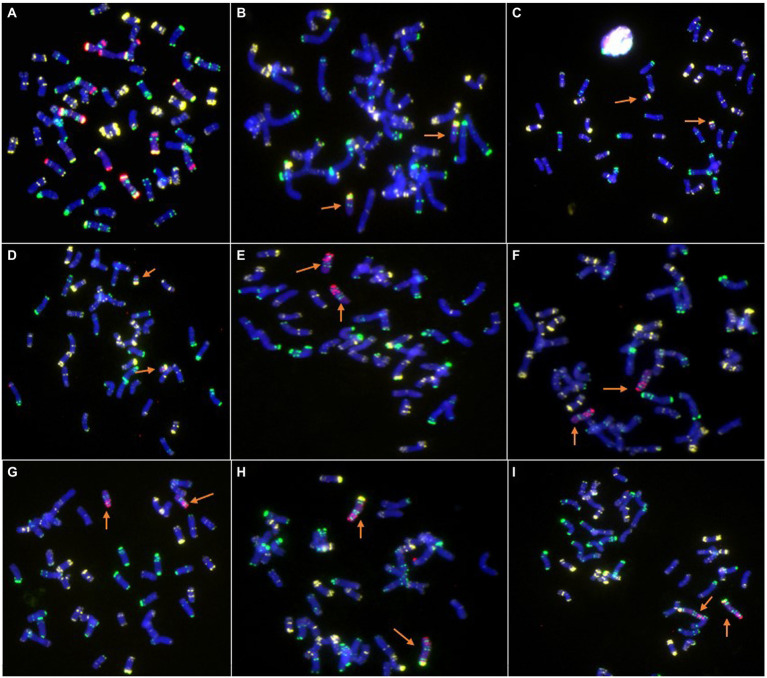
Cytogenetic analysis of new wheat-rye lines by ND-FISH. **(A)** Amphidiploid plants (2*n* = 56, 21″WW + 7″RR). **(B)** 46-13-3, 6RS ditelosomic addition line. **(C)** 46-13-11, 6RS ditelosomic addition line. **(D)** 46-13-16, 6RS ditelosomic addition line. **(E)** 48-9-2, 6RL ditelosomic addition line. **(F)** 48-9-5, 6RL ditelosomic addition line. **(G)** 48-9-6, 6RL ditelosomic addition line. **(H)** 48-9-8, 6R disomic addition line. **(I)** 48-9-10, 6R disomic addition line. The arrows show the 6R, 6RL, or 6RS chromosomes. Oligo-pSc119.2-1: green; Oligo-pTa535-1: white; Oligo-Ku, Oligo-pSc200, and Oligo-pSc250: red.

The pedigrees of the 6R disomic addition lines, 6RS ditelosomic addition lines, and 6RL ditelosomic addition lines are displayed in Figure 1. The wheat parent CN25 (2*n* = 42, 21″WW) was directly crossed with QL2 (2*n* = 14, 7″RR), and several F_0_ seeds (2*n* = 28, 21″W + 7″R) were obtained. The chromosome numbers of F_1_ plants were doubled by colchicine treatment to produce amphidiploid (C_0_) seeds (2*n* = 56, 21″WW + 7″RR; Figure 2A). The amphidiploid plants were then backcrossed with the wheat parent CN25 twice to produce the BC_2_F_1_ plants. The continued generations were reproduced by selfing, and only the plants that exhibited resistance to diseases were harvested and bred. In the BC_2_F_5_ generation, the chromosome composition of the plants was examined by ND-FISH during the seedling stage, and then, the plants were transplanted into the field. In this generation, several plants with rye 6R or partial 6R were identified, and these plants also showed resistance in the field. The seeds of these plants containing alien chromatin were harvested and reproduced by selfing. The seedlings of BC_2_F_6_ were also identified by ND-FISH. In this generation, several homozygous 6R disomic addition lines, 6RS ditelosomic addition lines, and 6RL ditelosomic addition lines were identified. On the other hand, several plants were heterozygous or missed the 6R chromosomes. When plants missed the 6R, they also lose disease resistance in the field. The homozygous addition lines were harvested and reproduced the next generation by selfing. The heterozygous plants were also harvested, and we continued to screen the homozygous plants in their progeny in the generation of BC_2_F_7_ to BC_2_F_8_. Finally, three 6RS ditelosomic addition lines (46-13-3, 46-13-11, and 46-13-16), three 6RL ditelosomic addition lines (48-9-2, 48-9-5, and 48-9-6), and two 6R disomic addition lines (48-9-8 and 48-9-10) were selected.

### Chromosome identification

The combination of the five probes Oligo-pSc119.2-1, Oligo-pTa535-1, Oligo-Ku, Oligo-pSc200, and Oligo-pSc250 can easily and accurately distinguish wheat and rye chromosomes in one cell. The ND-FISH results indicated that three lines, 46-13-3, 46-13-11, and 46-13-16, contained a pair of 6RS chromosome arms (Figures 2B–D). Three lines, 48-9-2, 48-9-5, and 48-9-6, contained a pair of 6RL chromosome arms (Figures 2E–G). Two lines, 48-9-8 and 48-9-10, contained a pair of 6R chromosomes (Figures 2H,I).

**Figure 3:**
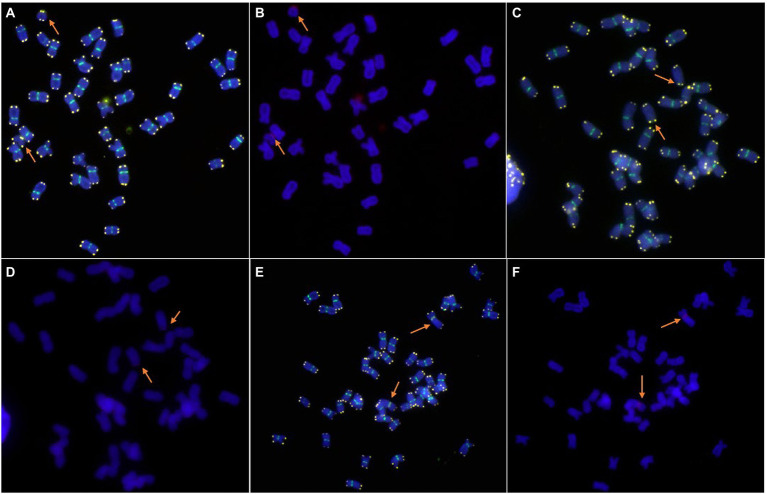
Cytogenetic analysis of the telomeres and centromeres of new lines by ND-FISH. **(A,B)**: 6RS ditelosomic addition line. The signal patterns were derived from one cell. **(C,D)**: 6RL ditelosomic addition line. The signal patterns were derived from one cell. **(E,F)**: 6R disomic addition line. The signal patterns were derived from one cell. Oligo-CCS1 (green), Oligo-PAWRC.1 (red), and Oligo-Telo (white). The arrows show the 6RS, 6RL, or 6R chromosomes.

The centromere and telomere structure of the new lines was identified using the combinations of the probes Oligo-CCS1, Oligo-PAWRC.1, and Oligo-Telo. The Oligo-CCS1 probe can detect both wheat and rye centromeres, whereas Oligo-PAWRC.1 can detect only rye centromeres. Therefore, the centromere of the 6R chromosome showed both signal patterns of Oligo-PAWRC.1 and Oligo-CCS1 (Figures 3E,F). The probe Oligo-Telo can detect the structure of the telomere and can also detect the telocentric chromosome. Therefore, the centromere of the 6RS and 6RL chromosome arms can show both signal patterns of the probes Oligo-PAWRC.1 and Oligo-Telo. The ND-FISH results showed that both 6RS and 6RL chromosomes showed complex signal patterns of Oligo-PAWRC.1 and Oligo-Telo (Figures 3A–D).

**Figure 4:**
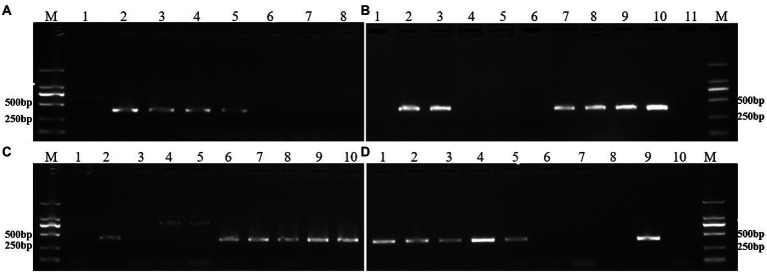
PCR results of specific molecular markers. **(A)** PCR results of the primer pair KU88. Lane 1: CN25, Lane 2: 46-13-3 (6RS), Lane 3: 46-13-11 (6RS), Lane 4: 46-13-16 (6RS), Lane 5: rye QL2, Lane 6: 48-9-2 (6RL), Lane 7: 48-9-5 (6RL), Lane 8: 48-9-6 (6RL). **(B)** PCR results of the primer pair KU291. Lane 1: CN25, Lane 2: 48-9-8 (6R), Lane 3: 48-9-10 (6R), Lane 4: 48-9-2 (6RL), Lane 5: 48-9-5 (6RL), Lane 6: 48-9-6 (6RL), Lane 7: 46-13-3 (6RS), Lane 8: 46-13-11 (6RS), Lane 9: 46-13-16 (6RS), Lane 10: rye QL2, Lane 11: CN25. **(C)** PCR results of the primer pair KU86. Lane 1: CN25, Lane 2: rye QL2, Lane 3: 46-13-3 (6RS), Lane 4: 46-13-11 (6RS), Lane 5: 46-13-16 (6RS), Lane 6: 48-9-2 (6RL), Lane 7: 48-9-5 (6RL), Lane 8: 48-9-6 (6RL), Lane 9: 48-9-8 (6R), Lane 10: 48-9-10 (6R). **(D)** PCR results of the primer pair KU153. Lane 1: 48-9-2 (6RL), Lane 2: 48-9-5 (6RL), Lane 3: 48-9-6 (6RL), Lane 4: 48-9-8 (6R), Lane 5: 48-9-10 (6R), Lane 6: 46-13-3 (6RS), Lane 7: 46-13-11 (6RS), Lane 8: 46-13-16 (6RS), Lane 9: rye QL2, Lane 10: CN25. Lane M: DNA marker DL2000. 6RS: 6RS ditelosomic addition lines; 6RL: 6RL ditelosomic addition lines; 6R: 6R disomic addition lines.

The chromosome composition of all lines was also confirmed by specific molecular markers. Two primer pairs, KU88 and KU291, were specific for the rye 6RS chromosome arm and amplified a specific band of ~400 bp. The PCR results showed that all DNA of the 6RS ditelosomic and 6R disomic addition lines amplified the expected bands at ~400 bp, whereas the bands were absent from the DNA of the 6RL ditelosomic addition lines and wheat CN25 (Figures 4A,B). On the other hand, the primer pairs KU86 and KU153 were specific for the rye 6RL chromosome arm and could also amplify a specific band at ~400 bp. The PCR results showed that all DNA of 6RL ditelosomic and 6R disomic addition lines can amplify the expected bands of ~400 bp, whereas the bands were absent from the DNA of 6RS ditelosomic addition lines and wheat CN25 (Figures 4C,D; Table 2).

### Resistance to stripe rust and powdery mildew

The wheat parent CN25 was intermediately resistant to two *Pst* races (CYR32 and 34) and highly susceptible to the *Bgt* isolate E20 and No. 15. CN25 also exhibited intermediate resistance to stripe rust and was highly susceptible to powdery mildew in the field (Table 3). On the other hand, inbred rye line QL2 was highly resistant to three *Pst* races and two *Bgt* isolates. QL2 also exhibited high resistance to stripe rust and powdery mildew in the field (Table 3). The control Mianyang 11 was highly susceptible to three *Pst* races and two *Bgt* isolates, and also exhibited susceptible to stripe rust and powdery mildew in the field. The control RT1104-1 exhibited resistance to three *Pst* races and two *Bgt* isolates, and also showed resistance in the field (Table 3). The 6R disomic, 6RS ditelosomic, and 6RL ditelosomic addition lines showed a high level of resistance to three *Pst* races and two *Bgt* isolates at the seedling stages. In addition, these eight lines also showed high resistance to stripe rust and powdery mildew in the field at the adult stage (Table 3).

**Table 3:** Analysis of resistance to stripe rust and powdery mildew when inoculated with prevalent races/isolates of stripe rust and powdery mildew.

Lines	Chromosome	*Pst* analysis				*Bgt* analysis		
		CYR32	CYR33	CYR34	In field	E20	No.15	In field
Chuannong 25	21″WW	4	3	5	5	4	4	4
46-13-3	21″WW + 1″6RS	0	0	0	0	0	0	0
46-13-11	21″WW + 1″6RS	0	0	0	0	0	0	0
46-13-16	21″WW + 1″6RS	0	0	0	0	0	0	0
48-9-2	21″WW + 1″6RL	0	0	0	0	0	0	0
48-9-5	21″WW + 1″6RL	0	0	0	0	0	0	0
48-9-6	21″WW + 1″6RL	0	0	0	0	0	0	0
48-9-8	21″WW + 1″6R	0	0	0	0	0	0	0
48-9-10	21″WW + 1″6R	0	0	0	0	0	0	0
Rye QL2	7″RR	0	0	0	0	0	0	0
Mianyang 11	21″WW	8	8	9	9	4	4	4
RT1104-1	T1RS.1BL	0	0	0	0	0	0	0

For *Pst* analysis: 0: no visible symptoms; 1: necrotic flecks; 2: necrotic areas without sporulation; 3: necrotic and chlorotic areas with restricted sporulation; 4–6: moderate uredinia with necrosis and chlorosis; 7–8: abundant uredia with chlorosis; 9: without chlorosis For *Bgt* analysis: 0: no visible symptoms; 0;: hypersensitive necrotic flecks; 1: minute colonies with few conidia produced; 2: colonies with moderately developed hyphae, but few conidia; 3: colonies with well-developed hyphae and abundant conidia, but colonies not joined together; 4: colonies with well-developed hyphae and abundant conidia, and colonies mostly joined together.

**Figure 5:**
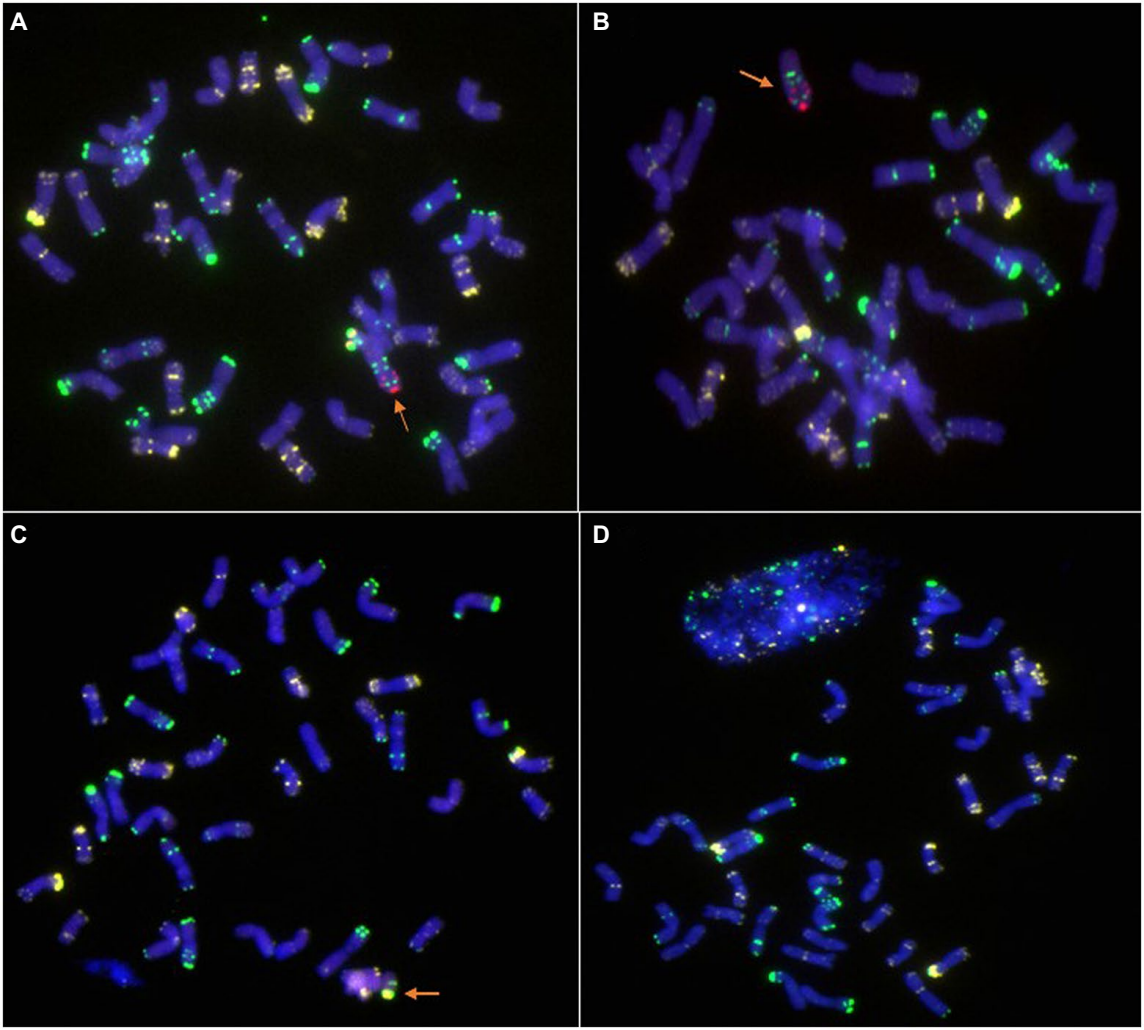
Lines with other types identified by ND-FISH. **(A)** 6R monosomic addition. **(B)** 6RL monotelosomic addition. **(C)** 6RS monotelosomic addition. **(D)** Lines that missed 6R. The arrows show the 6R, 6RL, or 6RS chromosomes. Oligo-pSc119.2-1: green; Oligo-pTa535-1: white; Oligo-Ku, Oligo-pSc200, and Oligo-pSc250: red.

Moreover, in addition to these homozygous 6R, 6RS, and 6RL disomic or ditelosomic addition lines, several other types, such as 6R monosomic addition (Figure 5A), 6RL monotelosomic addition (Figure 5B), and 6RS monotelosomic addition (Figure 5C), and several plants that missed 6R, 6RL, or 6RS (Figure 5D), were also identified in the generation of BC_2_F_7_–BC_2_F_8_. Without exception, plants exhibit disease resistance as long as they have 6R, 6RS, or 6RL in their genome, but disease resistance was lost when 6R, 6RS, or 6RL was missed (Table 4).

**Table 4:** Relationship between resistance and chromosome types.

Types	Chromosome	Resistance to *Pst*	Resistance to *Bgt*
6R disomic addition line	21″WW + 1″6R	0	0
6R monosomic addition line	21″WW + 1*′*6R	0	0
6RS ditelosomic addition line	21″WW + 1″6RS	0	0
6RS monotelosomic addition line	21″WW + 1*′*6RS	0	0
6RL ditelosomic addition line	21″WW + 1″6RL	0	0
6RL monotelosomic addition line	21″WW + 1*′*6RL	0	0
Lines missed rye chromatin	21″WW	5	4
CN25	21″WW	5	4

For *Pst* analysis: 0: no visible symptoms; 5: moderate uredinia with necrosis and chlorosis. For *Bgt* analysis: 0: no visible symptoms; 4: colonies with well-developed hyphae and abundant conidia, and colonies mostly joined together.

## Discussion

### 6R chromosome of Qinling rye provides resistance to *Pst* and *Bgt*

Related species of wheat are very important for the improvement of wheat genetics (Friebe et al., 1996), and rye is the most important species. According to Weining rye genome sequencing data, 1,909 disease resistance-associated (DRA) genes were identified in the rye genome (Li et al., 2021). Several useful resistance genes were introduced into the wheat genome through wheat-rye distance crosses. For example, *Yr9*, *Pm8*, *Pm17*, *Sr31*, and *Lr26* were derived from the 1RS chromosome arm through T1RS·1BL or T1RS·1AL chromosome translocation (Mago et al., 2005). *Sr59* and *H21* were derived from the 2RL chromosome arm through T2RL.2BS or T2RL.2DS chromosome translocation (Friebe et al., 1990; Rahmatov et al., 2016). *Sr27* was derived from the 3RS chromosome arm through the T3RS.3BL chromosome translocation (Marais and Marais, 1994). Many other rye chromosomes with resistance have been introduced into the wheat genome. For example, Ren et al. reported (Ren et al., 2020) a T7RL.7BS translocation line with resistance to *Pst*, *Bgt,* and *Fusarium* head blight. An et al. reported (An et al., 2019) a 4R disomic addition line with resistance to *Pst*, *Bgt*, and sharp eyespot. The 6R chromosome of rye also contains many excellent resistance genes and plays an important role in wheat genetic improvement. According to Weining rye genome sequencing data, 287 disease resistance-associated genes mapped to the 6R chromosome (Li et al., 2021). To date, stripe rust resistance gene *Yr83* has been mapped on the 6RL chromosome arm (Li et al., 2020), and powdery mildew stripe rust gene *Pm56* has been mapped on the 6RS chromosome arm (Hao et al., 2018). Several other 6R chromosomes have been introduced into the wheat genome. For example, Han et al. (2022) reported a 6R addition line originating from a cross between octoploid triticale and common wheat that exhibited resistance to powdery mildew. Duan et al. (2022) reported a T6RL.6BS translocation line with resistance to stripe rust and originating from rye AR106BONE. Du et al. (2018) reported a 6RL/6D small segment translocation line with powdery mildew resistance that originated from rye Kustro. Schneider et al. (2016) reported a 6R disomic addition line with resistance to stripe rust originating from *Secale cereanum*. An et al. (2015) reported a 6R disomic addition line with resistance to powdery mildew originating from the rye cultivar., German White. However, most of these new lines were resistant to only one disease. A few new lines exhibit resistance to both stripe rust and powdery mildew. In this study, three 6RS ditelosomic addition lines, three 6RL ditelosomic addition lines, and two 6R disomic addition lines were selected from the cross between wheat CN25 and rye QL2 (Figures 1–3). All eight new lines exhibited high resistance to both stripe rust and powdery mildew (Table 3). Once 6RS or 6RL chromosome arms were added to the wheat genome, resistance to stripe rust and powdery mildew was demonstrated. If 6R chromosomes were missed in the offspring, the resistance was also lost (Figure 5; Table 4). The results indicated that both the 6RS and 6RL chromosome arms of QL2 contained gene(s) associated with resistance to stripe rust and powdery mildew.

### Genetic diversity of rye resistance genes

Rye has great potential as a source for favorable genes, such as genes supporting higher yield, stress resistance, disease resistance, and other agronomic traits desirable for wheat genetic improvement (Ren et al., 2011). Rye is a wind-pollinated plant, and high levels of genetic diversity are found not only between cultivars originating from different regions but also within the same cultivar (Persson and von Bothmer, 2002; Ren et al., 2011). A large number of disease resistance genes may also be present in the rye genome (Ren et al., 2022). The results of genome sequencing of Weining rye also revealed that there were many DRA genes that existed in the rye genome and were distributed to seven chromosomes with NBS (nucleotide-binding site), RLK (receptor-like kinase), RLP (receptor-like protein), and CC-TM (coiled-coil plus transmembrane receptor) types (Li et al., 2021). The numbers of these DRA genes along the seven assembled chromosomes of Weining rye (1R to 7R) were 242, 296, 301, 301, 255, 287, and 227, respectively (Li et al., 2021). Considering the crucial importance of DRA genes in plant responses to biotic adversities (Kourelis and van der Hoorn, 2018; Li et al., 2021), they may facilitate efficient genetic studies and molecular improvement of disease resistance in wheat. For example, Ren et al. (2022) reported 166 different T1RS·1BL translocation lines, which originated from three rye cultivars. These T1RS·1BL translocation lines exhibited different resistance patterns when they were tested by different *Pst* races. The stripe rust resistance gene *Yr9* originated from Petkus rye (Mago et al., 2005). Shi et al. (2001) found an allele of *Yr9* that exhibited different resistance patterns to the tested *Pst* pathotypes from a T1RS·1BL line Weique, which also originated from Petkus rye. Ren et al. (2009) reported the *Pst* resistance gene *YrCn17* from the T1RS·1BL cultivar CN17, which originated from an inbred line of Petkus rye, L155. The powdery mildew resistance gene *Pm8* was derived from Petkus rye (Mater et al., 2004), and another *Bgt* resistance gene *Pm17* was also mapped on the 1RS chromosome of Amigo, which originated from rye Insave (Mater et al., 2004). Ren et al. (2009) also reported a *Bgt* resistance gene, *PmCn17,* from cultivar CN17. In recent years, more 1RS chromosomes have carried more disease-resistance genes into the wheat genome and played a positive role in wheat disease-resistance breeding. Similar to 1RS chromosomes, the other six chromosomes of rye (2R–6R) also contain a large number of DRA genes (Li et al., 2021), which has great potential in wheat disease-resistance breeding in the future. To date, the stripe rust resistance gene *Yr83* has been mapped to 6RL, and the powdery mildew resistance gene *Pm56* has been mapped to 6RS (Hao et al., 2018; Li et al., 2020). Obviously, there is also abundant genetic diversity on the 6R chromosome, which may contain more disease-resistance genes for wheat genetic improvement. In this study, six monotelosomic addition lines and two disomic addition lines were identified from the distance cross between rye line QL2 and wheat cultivar CN25. The resistance analysis showed that all new lines exhibited high resistance to *Pst* and *Bgt*. This finding indicated that similar to 1RS, high genetic diversity of resistance genes existed on the 6RS or the 6RL chromosome arms. The genetic diversity of resistance genes on alien chromosomes could be transferred into the wheat genome to increase the genetic diversity of resistance genes in wheat (Ren et al., 2022). Further utilization of the genetic diversity of resistance genes is an important method to further utilize rye resources to improve wheat genetics. These new lines could be used as a promising bridging parent and valuable genetic resource for wheat disease resistance improvement.

## Data availability statement

The original contributions presented in the study are included in the article/supplementary material, further inquiries can be directed to the corresponding authors.

## Author contributions

TR and ZL: conceived and designed the study. TR and ZR: created the materials. TR, ZR, and ZL: analyzed the data. TR: wrote the manuscript. ZS, YH, FT, and PL: performed the experiments and analyzed the data. All authors contributed to the article and approved the submitted version.

## Funding

This work was supported by the National Natural Science Foundation of China (31801357) and the Foundation of Sichuan Province Science and Technology Support Program (2019YJ0510, 2021YJ0509, and 2021JDRC0127).

## Conflict of interest

The authors declare that the research was conducted in the absence of any commercial or financial relationships that could be construed as a potential conflict of interest.

## Publisher’s note

All claims expressed in this article are solely those of the authors and do not necessarily represent those of their affiliated organizations, or those of the publisher, the editors and the reviewers. Any product that may be evaluated in this article, or claim that may be made by its manufacturer, is not guaranteed or endorsed by the publisher.
